# Cutaneous and Cerebral Metastases From Primary Peritoneal Clear Cell Carcinoma

**DOI:** 10.7759/cureus.21282

**Published:** 2022-01-16

**Authors:** Fátima R Alves, Mariana Malheiro, André Ferreira, Helena Miranda, Ana Martins

**Affiliations:** 1 Medical Oncology, Hospital de São Francisco Xavier, Centro Hospitalar de Lisboa Ocidental, Lisbon, PRT; 2 Medical Oncology, Hospital CUF Tejo, Lisbon, PRT

**Keywords:** central nervous system metastasis, brain metastases, skin metastases, cerebral metastases, cutaneous metastases, endometriosis, ovarian cancer, peritoneum, primary peritoneal carcinoma, clear cell carcinoma

## Abstract

Peritoneal tumors are very uncommon and among them, primary peritoneal clear cell carcinoma is extremely rare and often misdiagnosed as others subtypes. There are only 13 cases of primary peritoneal clear cell carcinoma previously reported in the literature and there are no reports about cutaneous metastasis in this setting and only brain metastases were described to be associated with other primary peritoneal carcinoma subtypes. More information about this topic is needed and so we are presenting a new case of primary peritoneal clear cell carcinoma with cutaneous and cerebral metastases in a 34-year-old female.

## Introduction

Peritoneum is a site for both primary and secondary tumors but primary peritoneal tumors are very rare, imposing diagnostic challenges for both clinicians and pathologists [1,2]. Primary peritoneal carcinoma is a rare extraovarian malignant tumor that originates from the epithelial cells of the abdominal/pelvic peritoneum [3]. The most frequently found neoplasms in the peritoneum are mesothelioma and epithelial tumors of Mullerian-derived just as serous papillary adenocarcinoma [3,4]. Clear cell carcinoma (CCC) is a Mullerian-type neoplasia and it is more frequent in the ovary and endometrium [1,4]. CCC exhibits a distinctive morphology, represented by wide tumor cells with abundant and clear cytoplasm and hobnail appearances, organized in different patterns such as tubulo-cystic, papillary or solid [1]. Primary peritoneal clear cell carcinoma (PPCCC) is an extremely rare medical condition considered a high-grade tumor and with poor prognosis [1,3]. Up to now, only 13 cases have been published in the Medical literature and as a result there is very limited data available about the biopathological characteristics of this entity, and the adequate patient approach and guidance [1,3].

Cutaneous metastasis is an uncommon manifestation of internal malignancy, occurring in 0.9% to 5.8% of patients with ovarian carcinoma [5-7]. Up to this date, to the best of our knowledge, there is no report of PPCCC with cutaneous metastasis. Central nervous system (CNS) metastasis is considered a rare occurrence in ovarian and peritoneal cancer, the incidence of which has been described as being between 0.3-12% in several studies [8]. The epidemiological data about brain metastasis of primary peritoneal carcinomas are scarce but the incidence seems to be similar or less than that for ovarian carcinoma [9].

Thus, we are presenting a new case of primary peritoneal clear cell carcinoma with cutaneous and cerebral metastases in a 34-year-old female.

## Case presentation

A 34-year-old Caucasian premenopausal woman was referred to our hospital in May 2019 with a diagnosis of primary peritoneum clear cell carcinoma made in December 2018. Her past medical and surgical history was unremarkable and the family history was negative for cancer. Menarche was at 9 years of age, she had one pregnancy and one abortion on her gynecological history and she was on oral contraception since 18 years of age.

**Figure 1:**
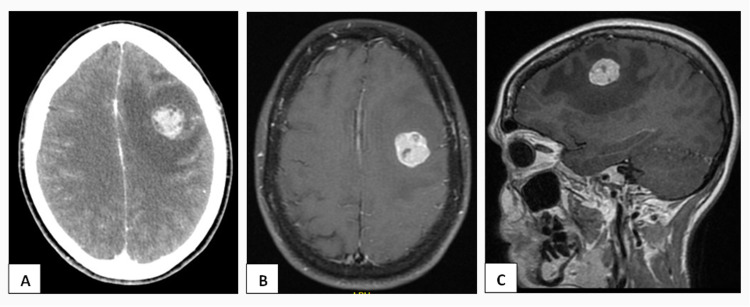
(A) Unique expansive lesion in the left cerebral hemisphere in CT scan (axial acquisition). (B) The same lesion in the brain MRI (axial acquisition) and (C) sagittal acquisition.

The initial complaints were persistent serous vaginal discharge and progressive abdominal discomfort for over the year. The gynecological evaluation detected a suspicious pelvic mass and the transvaginal ultrasound revealed an image suggestive of hydrosalpinx on the left (75x45 mm) and foci of adenomyosis and endometriosis. Both abdominal-pelvic computed tomography (CT) scan and pelvic magnetic resonance imaging (MRI) showed a large pelvic lesion measuring 8x8x5.5 cm extending from the pelvic floor (levator muscles of the anus) superiorly to the left ovary, compressing the vagina, rectum and left ureter with doubtful cleavage plane. The biopsy performed with ultrasound guidance was compatible with carcinoma of Mullerian origin and the initial FIGO (2014) staging was defined in IIB for ovarian carcinoma. The patient underwent a radical hysterectomy, anterior resection of the rectum, upper 1/3 of the vagina and left ureter in block (ureter reimplantation after resection), bilateral pelvic and interaortocaval lymphadenectomy, omentectomy and peritoneal lavage. Anatomopathological analysis revealed a high-grade primary carcinoma of the peritoneum of clear cell. The tumor involved the cervix, vaginal wall, rectal wall, bilateral parametrial and periureteral soft tissues, lymphatic and vascular invasion were present and pelvic lymph nodes were positive for cancer - metastases in four of 15 lymph nodes right pelvic, one of two promontory lymph nodes and 12 of 30 rectum lymph nodes. Endometriosis in the pouch of Douglas and surrounding the tumor was also present. The final pathological and clinical stage was defined: pT3apN1acM0 - IIIa2 (AJCC/TNM staging). The case was discussed at the multidisciplinary tumor board (MDTB) in our hospital and she was proposed to six cycles of adjuvant chemotherapy with Carboplatin AUC 5 + Paclitaxel 175mg/m2 (21/21 days). In the thoracic, abdominal and pelvic CT scan (TAP CT scan) assessment, there was no evidence of pelvic masses nor suspicious nodes but discreet amount of ascites in the pelvic cavity was detected and serum CA 125 was normal (13 U/mL). No relevant changes were detected in the pelvic MRI four months after the CT scan and the oncogenetic study revealed a variant of unknown clinical significance in the BRCA1 gene. One year after treatment, she presented with complaints of persistent headaches, nausea and vomiting and underwent a cranioencephalic CT scan that showed a single 22x20 mm left frontal lesion (Figure 1A). Brain MRI showed a 19x23x19 mm lesion with an extensive area of surrounding vasogenic edema in the left frontal cortico-subcortical intra-axial region (Figure 1B, 1C). The patient went through surgical excision of the tumor whose histopathology analysis was compatible with metastasis from clear cell carcinoma PAX8+ / WT1-.

TAP CT scan assessment presented with iliac and left inguinal adenopathies + small amount of fluid in the pelvic cavity. Left inguinal adenopathy was biopsied and was consistent with carcinoma (AE1/AE3+ e PAX8+) and serum CA 125 level was elevated at 84 U/mL. The patient began the first line of chemotherapy with Carboplatin AUC 5 + Paclitaxel 175mg/m2 and completed five cycles. Iliac and left inguinal adenopathies remained evident but with no appreciable changes in dimensions on the CT scan reassessment. Brain MRI (six months after brain surgery) showed three new intra-axial lesions: left parietal (1 cm), left frontal-basal (6 mm) and left superior frontal (6 mm). After discussion at the MDTB the patient was proposed to radiosurgery, however, radiotherapy planning CT scan revealed more extensive brain disease with a total of 16 small lesions, having begun IPARP (Poly-ADP-ribose polymerase inhibitors) at the time and undergoing holocranial radiotherapy (HC RT) with a total dose of 30 Gy/10fr (Image-guided radiation therapy).

During the four months after starting the IPARP, progression of the left inguinal adenopathies was evident on physical examination and also an increase in the serum CA 125 level - 132 U/mL. After completion of holocranial radiotherapy, the patient presented with disorientation and self-aggressiveness symptoms as a side effect of HC RT concomitantly with the use of corticosteroids, which motivated her admission to an acute palliative care unit. She remained hospitalized for two weeks with stabilizations of the clinical condition. However, due to grade 4 thrombocytopenia (6000 platelets), it was necessary to discontinue the IPARP. Upon discharge, it was possible to resume the IPARP (at a reduced dose) by normalizing the analytical values, and there were a few exudative skin lesions in the patient's anterior abdominal and inguinal regions. Mention should be made to the pneumomediastinum, diagnosed by CT scan after the patient complained of persistent odynophagia. This finding was interpreted by a thoracic surgery specialist as spontaneous possibly related to trauma during the psychotic episode that motivated the admission.

**Figure 2:**
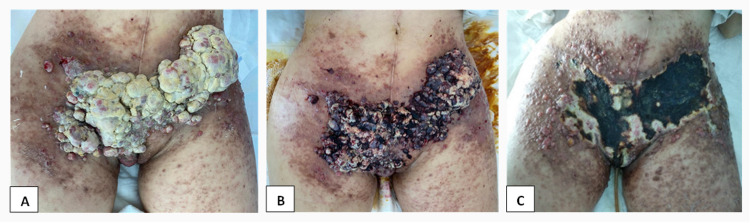
(A) Multiple cutaneous lesions in the abdomen. (B) Cutaneous lesions during electrochemotherapy. (C) Last electrochemotherapy session.

The patient kept outpatient follow-up by the support and palliative medicine team, as well as usual oncological appointments. From this moment, there was a rapid onset (progression in number and size) of the cutaneous exudative lesions in the anterior abdominal and inguinal region (Figure 2A), and as a result of severe asthenia and hematologic toxicity, the IPARP was definitely discontinued. Due to marked clinical and functional decline (ECOG PS 3) and the need for specialized care for pelvic/inguinal lesions due to bleeding, the patient was again admitted to the acute palliative care unit. There was a considerable blood loss from a major lesion on the external vaginal lip and putrid smell from the lesions, presence of superinfection and abundant exudate were observed. In addition to systemic support measures, for local control of the extensive skin disease, the patient underwent electrochemotherapy with an excellent clinical response (Figure 2B, 2C), resolving the blood loss and controlling the odor.

Despite effective local control, there was a systemic progression of the disease and the patient died two months after the palliative care unit admission, two years after the initial diagnosis and one year after the first relapse of the disease at 36 years of age.

## Discussion

Primary peritoneal tumors are infrequent and derive from the mesothelium or submesothelial membranes of the peritoneum [1]. Although rare, the most commonly seen malignant tumors of the peritoneum are malignant mesothelioma and serous papillary adenocarcinoma [2]. The present case is histologically different from these tumors, being PPCCC it is an exceedingly unusual subtype representing only 3% of all primary peritoneal neoplasia [2]. Differentiating tumors originating primarily from the peritoneum versus tumors originating from the ovary can be very difficult [1]. The Gynecology Oncology Group (GOG) criteria establish the diagnosis of primary peritoneal tumors as follows: “ovaries should be of normal size bilaterally or enlarged by benign processes plus the involvement in extraovarian locations should be larger than that on both ovaries”, this is originally described for primary adenocarcinoma of the peritoneum [1,10]. The pathological process of development of peritoneal serous carcinoma resembles to that of ovarian cancer and it may develop from the remnants of ovarian tissue in the peritoneum during embryonic development or from the mesoderm that originates the epithelium of the ovary and the peritoneum [10]. Therefore, pelvic/abdominal mesothelium may be the origin of primary peritoneal carcinoma similar to ovarian cancer and additionally, non-serous peritoneal carcinoma has been described, such as PPCCC [10]. The pathogenesis of non-serous carcinomas has been ascribed to the existence of an extraovarian extended Mullerian system or to peritoneal endometriosis. In particular, clear cell carcinoma of the ovary and those few reported primary extraovarian peritoneal carcinomas are often associated with endometriosis [11]. In the present case, our patient had evidence of endometriosis and adenomyosis on vaginal ultrasound and in the final histological analysis and, according to the previous 13 cases reported in the literature, approximately one-third had a previous gynecological record of endometriosis [1,3,10]. Endometriosis is one of the most common types of gynecologic disorders with an estimated incidence that ranges from 7% to 10% [4]. Malignant transformation in sites of endometriosis is an atypical occurrence, but the association between this pathology and the development of malignant tumors has been established [4]. The evidence available in the literature recognized that malignant tumors that grow at sites of endometriosis often exhibit clear cell or endometrioid histopathological features [1]. Plausible conjectures for the connection of these two lesions take into consideration a genetic defect in preexisting endometriosis and also a defect in the immune system that enables endometriosis to grow and expand and with an increased risk to malignant transformation [4].

Data on metastases in primary cancer of the peritoneum are very scarce and, considering the similarities with ovarian cancer, the existing information is mostly derived from extrapolation of the data on ovarian cancer. Ovarian carcinoma commonly metastasizes by direct extension from the ovarian tumor to adjacent pelvic organs or by transperitoneal dissemination of detached cancer cells [8]. Metastases through the blood vessels are less common [8]. In contrast, lymphatic metastasis is fairly frequent in both ovarian and peritoneal cancer as 70% of ovarian cancer women stage with pelvic and/or para-aortic lymph node involvement [8]. There are other rare distant locations that have been reported [8]. These can happen at the onset or during the evolution of the disease and they are frequently associated with hematogenous pathway and lymphatic invasion, with the less frequent locations being principally skin, bone, central nervous system (CNS), eye, breast, bronchus-trachea, heart-pericardium, rare lymph nodes and exceptionally unusual intra-abdominal sites, often with poor prognosis [8]. The reported incidence for CNS metastasis ranges widely from 0.3 to 12% with an average of 2% [8,12] and the incidence of skin metastasis varies from 0.9 to 5.8% [7,8].

CNS metastases mostly develop through hematogenous and lymphatic dissemination with reports of direct infiltration of the nervous tissue after bone involvement [8]. The spread pathways of primary peritoneal carcinoma, specifically, remain not so clear, but are believed to be the hematological and lymphatic pathways [9]. Primary peritoneal carcinoma metastasis to CNS could present as motor weakness, speech disturbance, seizures, mood alterations, memories disturbances, confusion and headaches as in the case reported [8,9]. These symptoms are present in almost 90% of patients at diagnosis. Nevertheless, CNS disease can be established in a number of patients without any neurological deficits [8]. Besides, corticosteroids and antiepileptics, surgical resection, radiotherapy and systemic chemotherapy constitute the best therapy to prolong the survival of these patients [8,13]. Particularly for patients with several cerebral metastases, whole-brain irradiation (+/- chemotherapy) prevails as the chosen treatment option, as proposed to our patient after the second CNS relapse [8]. In order to enhance the local and systemic control of the disease, the addition of chemotherapy drugs in particular carboplatin, cisplatin plus gemcitabine and carboplatin plus docetaxel, to local therapy, had been proposed in the literature [8].

To our knowledge, there are no cases of cutaneous metastasis of PPCCC described in the literature. This disease is extremely rare, and the majority of cases described in the gynecological setting are from ovarian cancer. Cutaneous metastatic ovarian carcinoma mostly occurs in the skin adjacent to the primary ovarian cancer including the abdominal wall and also in the skin of the chest and breast [6,8]. Based on the location of the lesion, cutaneous metastases are categorized into two groups: umbilical metastases known as Sister Joseph’s nodules (SJN) and metastatic non-umbilical tumors [7]. Non-umbilical (non-SJN) skin metastases often arise in recurrent stages and a variety of locations may be involved in non-umbilical relapses, being the abdominal surface the most commonly involved site [7]. The clinical signs of cutaneous metastases range widely and it can be characterized as a solitary or multiple skin nodules, grouped papules, herpetiform or inflammatory pattern nodules, as well as cicatricial plaques covering the entire surface of the abdomen [6,8]. Our patient presented, as described (Figure 2), with multiple non-umbilical nodules in the lower abdominal and inguinal regions. The precise mechanism of cutaneous metastases has not been entirely elucidated, but there are multiple hypotheses suggesting either direct invasion/implantation from inherent growth of the tumor, the lymphatic pathway or extra-nodular extension and hematogenous pathway [7,8]. Additional mechanism of metastases clearly stated in the literature is the iatrogenic one [8]. Skin metastases can develop at the site of the surgical incision, from accidental implantation of tumor cells during surgical procedures or from cicatricial tissue [7,8]. Contamination from port catheters, tissue manipulation, the use of paracentesis ascites catheters, and fine-needle biopsy has been shown to participate in the metastases [8]. In addition, cutaneous metastatic nodules can originate in the surrounding of the metastatic superficial nodes [7]. In the ovarian cancer setting, metastatic lesions in the lower abdominal wall and inguinal regions grow after metastatic inguinal node disease [7]. Our patient underwent multiple procedures such as a massive surgery and inguinal adenopathy biopsy that could justify the iatrogenic mechanism and superficial lymph nodes extension respectively. Besides that, direct invasion from primary peritoneal cells carcinoma is also a strong possibility considering the high-grade and aggressive clear cell histology. Furthermore, CCC is known for its chemorefractory feature and therefore it develops recurrences in surgical scars, even after undergoing adjuvant chemotherapy [7]. Similar to our patient's clinical evolution, cutaneous metastases after recurrence in other sites usually occur with other coexisting metastases [7]. The therapeutic options for patients with these skin recurrences are restricted, as they have already undergone multiple cycles of chemotherapy and have become chemoresistant [7]. Therapeutic options for metastatic non-umbilical lesion depend on the location of the disease, the existence or non-existence of concurrent diseases, and time to relapse. Surgical resection may be reasoned if the cutaneous lesion is an incisional metastasis without concomitant diseases and irradiation is effective to improve the symptoms related to the skin lesions and it may extend the longevity and quality of life in a number of patients [5]. Given the awareness of morbidity associated with cutaneous metastases, the interest in skin-directed therapies has surged. Electrochemotherapy (ECT) represents a valuable alternative/complementary option for patients with superficial tumors and compares very favorably with other skin-directed therapies [14]. Taking into account the multiple fast-growing skin nodules, our patient was a suitable candidate for ECT, with an excellent response to this treatment.

Both, CNS and cutaneous metastasis have a poor prognosis, with a median survival of 8.2 and 12 months, respectively [8]. Brain metastases result in a significant reduction in quality of life, however, brain metastases are heterogeneous in terms of the type of primary tumor, the extent of disease, therapeutic sensitivity and general condition of a patient [15]. In general, the prognosis of patients with cutaneous metastases is perceived to be considerably poor, given the fact that these types of metastases usually appear late in the progression of the disease at an advanced stage particularly in non-umbilical metastases [8]. In the presented case, the patient died 12 months after the first CNS recurrence (six months after the second) and three months after the cutaneous metastasis.

## Conclusions

Primary clear cell carcinoma of the peritoneum is rare and there are, to date, a small number of cases described in the literature. Although PPCCC is considered extremely rare, its acknowledgement is essential for adequate evaluation and management. We presented a distinctive case of a PPCCC with CNS and cutaneous metastasis. Due to the rarity of the reports, these rare sites are not fully exploited, representing a challenge in clinical practice. Adequate treatment should be individualized and chosen based on the patient, the presence or absence and location of concomitant metastatic disease. Taking into account the morbidity associated with this rare histologic subtype, more studies are required to further understand the specific biology, prognosis and to outline more effective management approaches.
